# Long-term fracture risk in rheumatoid arthritis: impact of early sustained DAS28-remission and restored function, progressive erosive disease, body mass index, autoantibody positivity and glucocorticoids. A cohort study over 10 years

**DOI:** 10.1186/s41927-023-00347-6

**Published:** 2023-08-07

**Authors:** Sofia Ajeganova, Maria Andersson, Kristina Forslind, Inger Gjertsson, Britt-Marie Nyhäll-Wåhlin, Björn Svensson, Ingiäld Hafström

**Affiliations:** 1 Division of Gastroenterology and Rheumatology, Department of Medicine Huddinge, Karolinska Institutet, Stockholm, Sweden; 2 Department of Clinical Sciences, Rheumatology Division, Universitair Ziekenhuis Brussel, Vrije Universiteit Brussel, Laarbeeklaan 101, Brussels, Jette, 1090 Belgium; 3 Faculty of Medicine, Department of Clinical Sciences Lund, Lund University, Rheumatology, Lund, Sweden; 4 Spenshult Research and Development Center, Halmstad, Sweden; 5 Department of Rheumatology and Inflammation research, Institute of Medicine, Sahlgrenska Academy, Gothenburg University, Gothenburg, Sweden; 6 Department of Rheumatology, Falun Hospital, Falun, Sweden; 7 Rheumatology Unit, Karolinska University Hospital, Stockholm, Sweden

**Keywords:** Early rheumatoid arthritis, Fracture risk, Bone mineral density, Body mass index, Sustained disease control, Functional ability, Autoantibody, Erosive disease

## Abstract

**Background:**

Risk of fragility fractures in patients with rheumatoid arthritis (RA) is increased. Disease-related inflammation in RA is associated with low Bone Mineral Density (BMD). However, effects of specific disease factors on fracture occurrence and whether or not such disease effects are independent of BMD are unknown.

**Methods:**

Analysis of fracture outcome in the prospective cohort of 2557 patients with early RA (67% women, mean age 58.1 ± 15.6 years) during an observation period of 10.6 ± 4.7 years. In 602 patients BMD was measured at baseline. The first major fragility fractures were considered. Kaplan-Meier and Cox regression analysis, adjusted for traditional factors, prior fracture, disease activity and period of inclusion, were used to estimate the risk of the outcome.

**Results:**

During follow-up fracture occurred in 352 patients (13.8%), a rate of 13/1000 p-y. A proportional risk reduction for the outcome was associated with Body Mass Index (BMI) at baseline, BMI ≥ 30 kg/m^2^, and over the first two years sustained Disease Activity Score (DAS28)-remission, DAS28-low disease activity and Health Assessment Questionnaire (HAQ) ≤ 0.5. The proportional risk elevation for fractures was associated with BMI ≤ 20 kg/m^2^, DAS28 at baseline, 6-month and at 1-year, cumulative DAS28 over the two years, RF, erosion score progression at 2-year, HAQ score and HAQ ≥ 1 at 6-month and 1-year and showed a trend for ACPA positivity. The estimated fracture risk was increased in users of glucocorticoids (GC), associated with a higher GC-dosage at follow-ups and a higher cumulative dosage over two years, independently of disease activity. With adjustment for BMD, there was no difference in fracture outcome by exposure to GC. The effects of a higher BMI, DAS28-remission and low HAQ ≤ 0.5 attained at 6-month of treatment initiation and sustained up to 2 years, RF, ACPA, and erosion score progression at 2-year were independent of low BMD.

**Conclusions:**

This analysis supports importance of RA-specific risk factors in early RA for future major fragility fractures. Treat-to-target strategy and restored functional capacity in early RA-disease are important to prevent fractures. Autoantibody positivity, progressively erosive disease, and low weight could have additional value for personalized fracture preventive strategies in early RA.

## Background

Rheumatoid arthritis (RA) is the most common chronic systemic autoimmune disease, characterized by arthritis of peripheral joints. The incidence of RA increases with age, with a peak incidence in the 7th decade of life both in women and men [1]. One of the extra-articular complications of RA is bone remodeling characterized by an increase in bone resorption and a decrease in bone formation, causing a local peri-articular bone loss, joint erosions and generalized bone loss. Generalized bone loss may lead to secondary osteoporosis. The major clinical consequence of osteoporosis is the occurrence of fragility fractures with a significant impact on suffering, poor functional status, and mortality, which adds to the overall burden of RA.

RA is included in the 10-years FRAX (Fracture Risk Assessment tool) fracture risk calculator but, as a dichotomous predictor, it disregards the complexity of RA [2]. The pathophysiology of bone loss and secondary osteoporosis in RA is multifactorial and attributed to general factors such as higher age, female sex, low body mass index (BMI), RA-disease characteristics and treatments, in particular the use of glucocorticoids (GC) [3]. Bone loss starts already early in the disease course [4] and increases with longer disease duration [5]. The prevalence of bone loss and the risk of osteoporosis-related fragility fractures (both vertebral and non-vertebral) is about double that expected in non-RA populations [6–8]. Despite recent therapeutic advances, the risk of fragility fractures in RA is still increased compared with that in the general population [8, 9]. Predicting the risk of fragility fractures in RA remains, therefore, of utmost importance to optimize prevention strategies.

In daily practice, assessment of bone mineral density (BMD) is recommended to determine bone quality, but is insufficient to estimate fracture risk in individual patients because many fractures occur in the osteopenic range of BMD [10]. The results of the available studies on bone quality and fracture risk in RA vary by the type of fracture predicted and the bone compartment measured, have a number of important limitations such as cross-sectional design, exclusion of males, use of bone formation markers and BMD as surrogate outcomes, and omission of important disease characteristics. Furthermore, and only few prospective studies have been performed in early RA [11–15].

In the present study we aimed (i) to examine the relationship between several risk factors during the first two years of early RA disease and occurrence of major fractures, and (ii) to clarify whether or not the identified effects are independent of BMD i.e. if these factors could add value beyond BMD in the evaluation of fracture risk.

## Methods

### Patients and outcome assessment

We used a well-characterised early RA cohort, BARFOT (Better Anti-Rheumatic PharmacO Therapy), including consecutive adult patients newly diagnosed with RA according to the 1987 ACR criteria [16]. At inclusion, the patients had symptom duration ≤ 1 year and were naïve to glucocorticoids (GCs) and disease-modifying anti-rheumatic drugs (DMARDs). This prospective observational patient population comes from both urban and rural referral areas of six secondary care rheumatology units in the southern Sweden and Stockholm. All patients with available data were eligible for this study. The patients were enrolled between 1992 and 2006. For a further cohort description see elsewhere [17].

The study was approved by the local ethic committees. All patients gave their informed consent to the study.

### Outcome assessment

The outcome of the study was the first fragility fracture during the observation period. The fractures considered were traditional major fragility fractures such as fractures of the proximal femur (hip), thoracic/lumbar vertebrae (spine), proximal humerus (shoulder) and distal forearm (wrist). Fracture data were identified through medical chart reviews and resources of the Swedish national Registries such as the Hospital Discharge Registry and the Outpatients Register from 1987 to 2012, and the National Cause of Death Registries through 2012. The following International Classification of Diseases (ICD)-9 or ICD-10 codes were used: 820, S72 (hip fractures), 805, S22, S32, M48.5 (spine), 812, S42 (shoulder), and 813, S52, S62 (wrist fractures). Traumatic fractures due to accidents were not considered.

The time at-risk started between 1992 and 2006 when the patients were enrolled in the BARFOT-cohort. Patients were followed until the occurrence of the first major fracture (index fracture), or death, or censoring date of December 2012, whichever occurred first.

### Patients, risk factors and RA-disease characteristics

Demographics and information on treatments were obtained from the BARFOT database. Information on body weight and height at inclusion was extracted from the BARFOT database and medical records. BMI was calculated as weight/height² (kg/m²), and low weight was defined if BMI ≤ 20 kg/m^2^, normal weight if 20 < BMI < 25 kg/m^2^, overweight if 25 ≤ BMI < 30 kg/m^2^, and obesity if BMI ≥ 30 kg/m^2^ [18].

The following common risk factors were registered and confirmed through medical chart reviews: smoking history, hypertension (self-reported history of hypertension, hypertension by medical chart review, prescription of anti-hypertensive drugs, or blood pressure ≥ 140/90mmHg), diabetes mellitus (prescription of anti-diabetic drugs), dyslipidemia (medication prescription), cardiovascular disease (CVD) (coronary heart disease, ischemic cerebral and vascular disease), osteoporosis (osteoporosis by medical chart review or registry data, medication prescription, T-score < -2.5 SD).

Because of frequent prescription of GC at inclusion, patients were invited to undergo dual-energy x-ray absorptiometry (DXA) with a Lunar densitometer (Lunar Corporation, Expert-XL software version 1.7, 1998). The BMD was expressed as the number of standard deviations (SD) from the mean of healthy, young sex-matched people, i.e. T-scores, values obtained from Lunars combined European/US reference population. The lowest T-scores at hip (total hip and femoral neck) and at lumbar spine (L1-L4; antero-posterior view) were registered. Normal BMD was defined as a T-score more than − 1.0 SD, osteopenia if a T-score − 1.0 to -2.5 SD, and osteoporosis if a T-score was − 2.5 SD or less, according to the World Health Organisation criteria for osteoporosis [19].

In the BARFOT program the RA-disease characteristics were assessed at predefined intervals. For the purposes of this study the first two years of follow-up were considered. The disease activity was calculated using the Disease Activity Score for 28 joints (DAS28) with erythrocyte sedimentation rate (ESR) [20]. Remission was defined as DAS28 ≤ 2.6, and low disease activity (LDA) as DAS28 ≤ 3.2 [21].

Functional status was self-assessed by the validated Swedish version of the Stanford Health Assessment Questionnaire (HAQ), range 0 to 3 [22]. The HAQ values were categorized in the disability groups: HAQ ≤ 0.5 (normal) and HAQ ≥ 1 (disabled) [23, 24]. Remission, LDA and disability were defined as sustained when the respective DAS28 and HAQ values were reached and maintained at all follow-up assessments at 6-month, 1- and 2-year. Cumulative DAS28 and dose of GC used during the first 2 years after inclusion were calculated using the trapezoidal rule of area under the curve (AUC) based on data at inclusion and the assessments at 3, 6, 12 and 24 months.

Disease severity measures further included presence of autoantibodies and radiographic changes. Rheumatoid factor (RF) and anti-citrullinated protein antibodies (ACPA) were measured according to laboratory standards at the participating hospitals. Posterior-anterior radiographs of hands and feet were taken at inclusion and after 1 and 2 years and scored by two readers using the Sharp-van der Heijde scoring (SHS) method [25]. The between reader intraclass correlation coefficient (ICC) was 0.940 and the within reader ICC 0.998. The progression in SHS at 1- and 2-year was defined as a change ≥ 1 SHS-unit in comparison with the baseline reading.

Treatment was started and adjusted during follow-up according to routine care and the rheumatologists’ judgments. The treatment strategies that were usually applied changed over time, from GC, NSAIDs and “step-up” therapy in 1992–1995, towards mostly mild DMARDs in 1995–1999, and early introduction of methotrexate (MTX) and combination therapy including biologics in 2000–2006. These inclusion periods were used as a proxy for changes in treatment strategy.

### Statistical methods

Descriptive statistics are reported as means (SD) for continuous and percentages for categorical variables. To compare variables between the groups with/without fractures one-way ANOVA and Mann-Whitney test for independent samples and chi-square test was used as suitable.

Rates of event-free survival in patients with and without fractures were compared using Kaplan-Meier analysis. Equality of time-to-event function between the groups was tested with log-rank test. Relative hazard ratios from Cox proportional-hazards regression models were used to estimate the effect of risk factors on the fracture outcome. Into the multivariate models we entered known risk factors for fractures (age, sex, BMI, smoking, prior fracture, osteoporosis), the variables which distribution were different by the outcome at the level of significance p < 0.1 (hypertension and DAS28 at baseline) and an inclusion period. Effect sizes are reported as hazard ratio (HR) with 95% CIs. Due to possible differences in how data on fractures were registered over time, we performed sensitivity analyses of the main findings separately on the outcome of a hip fracture, because the level of completeness of registry data on acute femoral fractures in the inpatient (hospital) and outpatient specialist care has been estimated as high as 97% [26].

Significance tests were two-tailed and conducted at the 0.05 level of significance. IBM SPSS, version 27 (SPSS Inc., Chicago IL) was used for the analyses.

## Results

In all, 2557 patients with early RA were followed for 10.6 ± 4.7 years (median 10.3, IQR 7.6–14.3). Three hundred fifty-two patients (13.8%) occurred with fracture outcome (the rate of 13/1000 p-y). The site of index fracture was hip n = 152, spine n = 21, shoulder n = 63, wrist n = 101, and 15 patients had index fractures at several sites (10 hip and wrist, 3 shoulder and wrist, 1 spine and shoulder, 1 hip and shoulder).

### Characteristics of patients by fracture outcome

Table 1 presents the characteristics of the 2557 patients with RA, separately for the 352 patients who experienced a fracture during follow-up and the 2205 patients who did not. Of all, 5.2% of the patients had a fragility fracture prior to the date of the RA diagnosis. Patients with fractures during the study, compared with those without, were more likely to be older, females, and more likely to have a lower BMI, prior fractures, osteoporosis, and hypertension, but not dyslipidaemia, diabetes mellitus or CVD. Patients with fractures were also more likely to be underweight at baseline (BMI ≤ 20 kg/m^2^) than patients without fractures (12.3% versus 5.5.%, p < 0.001), whereas the patients without fractures were more likely to be obese (BMI ≥ 30 kg/m2) (13.2% versus 5.9%, p < 0.001).

**Table 1:** Characteristics of 2557 patients with early RA, of whom 352 occurred with fracture

	Total (n = 2557)	With fracture (n = 352)	No fracture (n = 2205)	P
Age, years	58.1 ± 15.6	66.3 ± 12.5	56.7 ± 15.7	**< 0.001**
Female sex, %	67.0	77.1	65.4	**< 0.001**
Current smoking, %	26.8	26.2	26.9	0.805
Ever smoking, %	59.7	60.5	59.5	0.728
Body mass index, kg/m^2^	25.3 ± 4.2	24.4 ± 3.8	25.5 ± 4.2	**< 0.001**
BMI ≤ 20 kg/m^2^, %	6.4	12.3	5.5	**< 0.001**
20 < BMI < 25 kg/m^2^, %	47.1	46.5	47.1	0.836
25 ≤ BMI < 30 kg/m^2^, %	34.4	35.3	34.2	0.726
BMI ≥ 30 kg/m^2^, %	12.2	5.9	13.2	**< 0.001**
Prior fractures, %	5.2	9.1	4.6	**< 0.001**
Osteoporosis, %	32.0	64.7	23.6	**< 0.001**
Hypertension, %	11.6	14.7	11.1	**0.046**
Dyslipidemia, %	2.6	2.9	1.3	0.264
Diabetes mellitus, %	5.5	5.7	4.2	0.262
CVD, %	9.5	11.6	9.2	0.153
**RA-disease dependent risk factors**				
Symptom duration at diagnosis, months	6.0 ± 3.2	5.8 ± 3.5	6.1 ± 3.2	0.129
Autoantibody positivity, %	73.2	74.9	72.9	0.461
RF-positivity, %	61.0	64.3	60.5	0.172
ACPA-positivity, %	56.8	58.3	56.6	0.596
DAS28				
at diagnosis	5.3 ± 1.2	5.5 ± 1.3	5.2 ± 1.2	**0.004**
at 6-month	3.5 ± 1.4	3.7 ± 1.4	3.5 ± 1.4	**0.006**
at 1-year	3.3 ± 1.4	3.6 ± 1.5	3.3 ± 1.4	**0.002**
at 2-year	3.2 ± 1.4	3.3 ± 1.3	3.1 ± 1.4	**0.048**
DAS28 ≤ 2.6 at 6-month, %	29.6	23.9	30.5	**0.019**
DAS28 ≤ 2.6 at 1-year, %	34.7	30.7	35.3	0.104
DAS28 ≤ 2.6 at 2-year, %	39.9	33.3	40.9	**0.012**
DAS28 ≤ 2.6 sustained, %	17.2	11.0	18.2	**< 0.001**
DAS28 ≤ 3.2 at 6-month, %	44.2	37.5	45.3	**0.011**
DAS28 ≤ 3.2 at 1-year, %	50.3	44.0	51.2	**0.017**
DAS28 ≤ 3.2 at 2-year, %	55.5	53.5	55.8	0.443
DAS28 ≤ 3.2 sustained, %	29.6	22.6	30.7	**0.003**
HAQ				
at baseline	1.1 ± 2.1	1.1 ± 0.7	1.1 ± 2.3	0.561
at 6-month	0.6 ± 0.6	0.7 ± 0.6	0.6 ± 0.6	**0.005**
at 1-year	0.6 ± 0.6	0.7 ± 0.6	0.6 ± 0.6	**0.014**
at 2-year	0.6 ± 0.6	0.7 ± 0.7	0.6 ± 0.6	**< 0.001**
HAQ ≤ 0.5 at 6-month, %	52.5	45.8	53.6	**0.011**
HAQ ≤ 0.5 at 1-year, %	55.3	50.6	56.0	0.075
HAQ ≤ 0.5 at 2-year, %	55.2	45.0	56.8	**< 0.001**
HAQ ≤ 0.5 sustained, %	39.4	32.0	40.6	**0.003**
HAQ ≥ 1 at 6-month, %	28.8	36.6	27.6	**0.001**
HAQ ≥ 1 at 1-year, %	27.4	33.9	26.4	**0.006**
HAQ ≥ 1 at 2-year, %	26.8	31.7	26.1	**0.042**
HAQ ≥ 1 sustained, %	12.2	14.8	11.7	0.112
**Radiographic RA-disease characteristics**				
SHS score at baseline	1 (0–6)	2 (0–8)	0.5 (0–6)	**0.005**
Erosion score at baseline	0 (0–1)	0 (0–2)	0 (0–1)	0.454
JSN score at baseline	0 (0–4)	1 (0–6)	0 (0–4)	**0.003**
SHS score at 2-year	5 (0–17)	8 (1.8–21.8)	5 (0–17)	**< 0.001**
Erosion score at 2-year	1.0 (0-5-25)	1 (0-6.88)	1 (0–5)	**0.041**
JSN score at 2-year	3 (0–11)	6 (0–14)	3 (0–11)	**< 0.001**
SHS progression at 2-year, %	60.4	66.1	59.5	**0.036**
Erosion progression at 2-year, %	47.1%	55.4	45.8	**0.004**
JSN progression at 2-year, %	48.9	55.1	47.9	**0.024**
**RA-treatments**				
Daily prednisolone use, %				
at baseline	41.9	45.8	41.2	0.113
6-month	40.4	43.1	39.9	0.287
1-year	35.8	40.9	35.0	**0.038**
2-year	31.1	36.3	30.2	**0.031**
Daily prednisolone dosage (only users), mg				
at baseline	7.5 (0–10)	7.5 (2.5–10)	7.5 (0–10)	0.869
6-month	5 (0-7.5)	5 (0-7.5)	5 (0-7.5)	0.768
1-year	3.75 (0-7.5)	5 (0-7.5)	3.75 (0-7.5)	0.109
2-year	2.5 (0–5)	2.5 (0–5)	2.5 (0–5)	0.212
AUC of prednisolone dosage over 1 year, mg/month	28.7 ± 37.7	32.5 ± 41.0	28.1 ± 37.1	**0.042**
AUC of prednisolone dosage over 2 years, mg/month	50.0 ± 66.2	57.0 ± 71.7	48.8 ± 65.2	**0.031**
Methotrexate over 2 years, %	63.6	64.1	63.6	0.853
Combined cDMARDs over 2 years, %	17.4	17.3	17.4	0.964
Biological agent over 2 years, %	7.0	5.1	7.4	0.125

Values are mean ± SD or median (IQR) or %

p-value (in bold if < 0.5) represents the comparisons between the patients who experienced a fracture and those who did not

BMI, body mass index; CVD, cardiovascular disease; RF, rheumatoid factor; ACPA, anti-citrullinated protein antibodies; DAS28, 28-joint disease activity score; HAQ, Health Assessment Questionnaire; SHS, Sharp-van der Heijde score; JSN, joint space narrowing; AUC, area under the curve; cDMARDs, conventional disease-modifying anti-rheumatic drugs

Sustained: indicated values maintained at the assessments of 6-month, 1-year and 2-year

Progression: progression of a score from baseline ≥ 1 unit

In terms of RA-specific risk factors, the patients with fractures had a more active arthritis at baseline and persistently through the two years, although the frequency of RF and ACPA positivity was similar in patients with or without a fracture. The patients who experienced a fracture, compared with those who did not, were less likely to achieve DAS28 remission or DAS28 low disease activity at follow-ups, and were less likely to to achieve a sustained DAS28 remission or DAS28 low disease activity at 2-year.

The patients who experienced a fracture and those who did not, had a similar level of disability at baseline, but patients who experienced a fracture were more likely to have a higher HAQ score at follow-ups and they were more often persistently disabled (HAQ ≥ 1) (Table 1).

In accordance with an older age at baseline, the joint space narrowing (JSN) score was higher in patients who developed a fracture compared with those who did not, but there was no difference in erosion score. However, at 2-year, both JSN and erosion scores were more likely to increase in patients with a fracture (55.1% versus 47.9%, p = 0.024, and 55.4% versus 45.8%, p = 0.004, respectively).

Almost one-half of the patients (41.9%) started treatment with daily prednisolone at baseline. While exposure to daily prednisolone decreased to 31.1% at the 2-year follow-up in this cohort, patients with a fracture, compared with those without, were more likely to be on prednisolone at 1- and 2-year follow-up (40.9% versus 35.0% and 36.3% versus 30.2%, p < 0.05) and were cumulatively exposed to a higher dose of prednisolone over 2 years (57.04 ± 71.70 versus 48.84 ± 65.18 mg/months, p = 0.031). The majority of RA patients were treated with MTX (63.6%) over the course of the two years after diagnosis. Exposure to MTX, combination of conventional DMARDs and biological agents was not different between those with a fracture or without.

### Risk factors for fracture

**Fig. 1:**
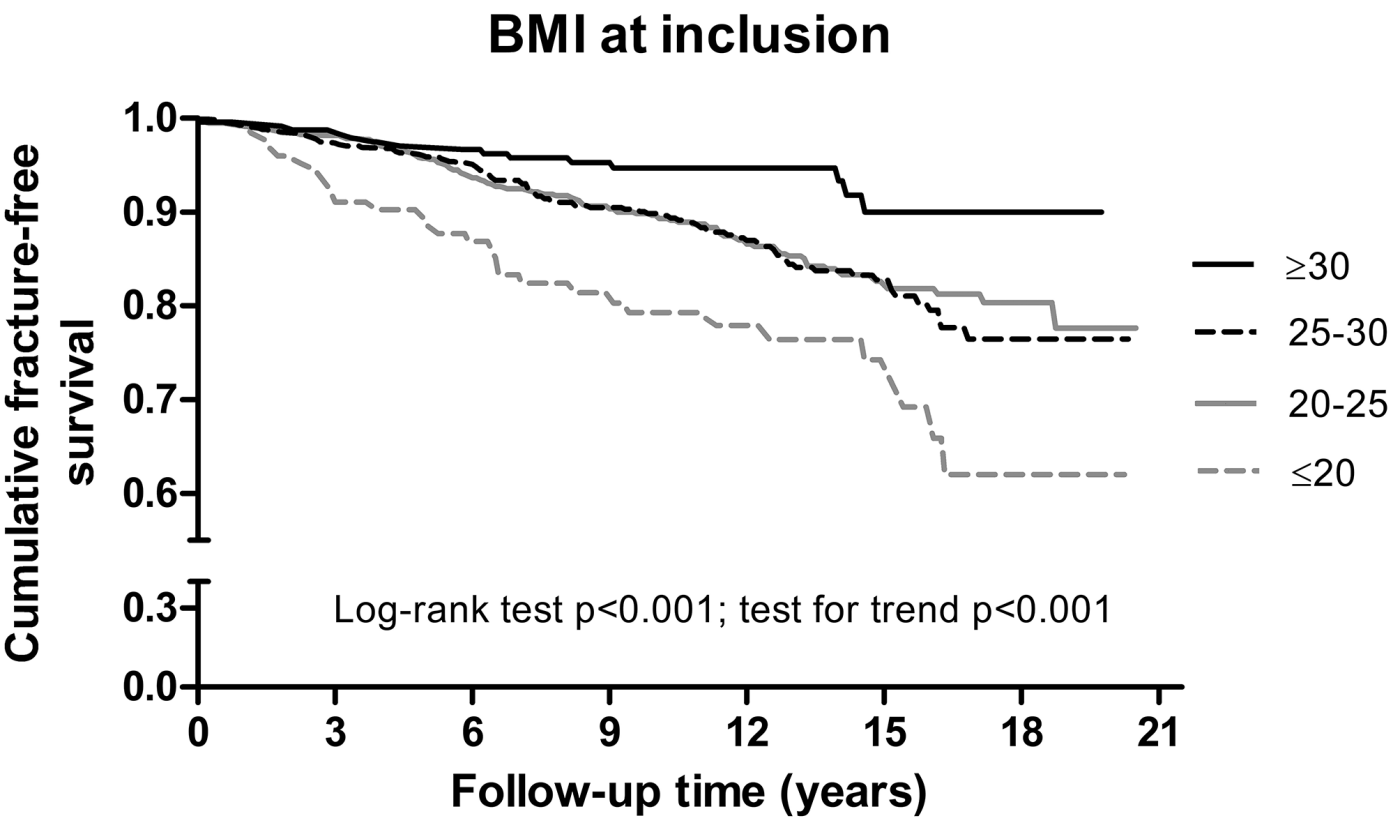
Kaplan-Meier curves of fracture-free survival in RA patients according to body mass index. Fracture occurrence by body mass index (BMI) at inclusion: of 1717 patients with available BMI data fractures occurred in 31 out of 94 patients with BMI ≤ 20 kg/m^2^, in 121 out of 818 patients with 20 < BMI < 25 kg/m^2^, in 95 out of 587 patients with 25 ≤ BMI < 30 kg/m^2^, and in 15 out of 226 patients with BMI ≥ 30 kg/m^2^.

**Fig. 2:**
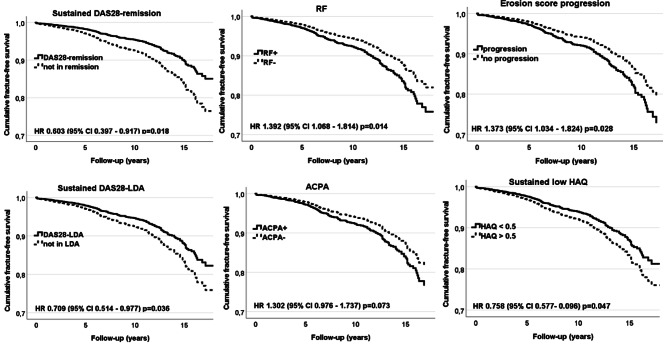
Cox proportional regression fracture-free survival in early RA patients according to risk factors. Illustrates Cox regression curves adjusted for age, sex, period of inclusion, BMI, smoking, prior fracture, osteoporosis, hypertension, DAS28 at baseline or a cumulative DAS over first 2 years after inclusion. HR, hazard ratio; sustained = if values were reached and maintained at follow-up at 6-month, 1- and 2-year; DAS28-remission = DAS28 ≤ 2.6; DAS28-LDA = DAS28 low disease activity if DAS28 ≤ 3.2; RF, rheumatoid factor; ACPA, RF, rheumatoid factor; ACPA, anti-citrullinated protein antibodies; low HAQ = HAQ ≤ 0.5; erosion score progression = if a change ≥ 1 unit at 2-year in comparison with baseline

Hazard ratios (HRs) for fractures are presented in Table 2 and the main results are illustrated in Figs. 1 and 2. After adjusting for age, sex, BMI, smoking, prior fracture, osteoporosis, hypertension, DAS28 at baseline or cumulative DAS28 and period of inclusion, the proportional risk reductions for major fractures were associated with BMI ≥ 30 kg/m^2^ at baseline, attained DAS28-remission or DAS28-LDA at 6-month of treatment initiation and sustained up to 2 years, HAQ ≤ 0.5 at 2-year, and attained HAQ ≤ 0.5 at 6-month of treatment initiation and sustained up to 2 years. The proportional risk elevation for fractures was associated with BMI ≤ 20 kg/m^2^ and DAS28 at baseline, DAS28 at 6-month and at 1-year, DAS28-AUC over the 2 years, the HAQ score and HAQ ≥ 1 at 6-month and at 1-year.

**Table 2:** Hazard ratios (95% CI) for fractures after RA onset by risk factors

Risk factor	Unadjusted HR	Age and sex adjusted HR	Multiple adjusted HR^a^
Age, years	1.07 (1.06–1.08)	1.07 (1.06–1.08)	1.06 (1.05–1.08)
Female sex	1.62 (1.27–2.08)	2.07 (1.62–2.66	1.67 (1.23–2.26)
Current smoking	0.97 (0.76–1.23)	-	-
Body mass index	0.94 (0.91–0.97)	0.91 (0.88–0.95)	0.92 (0.89–0.95)
BMI ≤ 20 kg/m^2^	2.16 (1.50–3.10)	2.72 (1.88–3.93)	2.40 (1.62–3.54)
20 < BMI < 25 kg/m^2^	0.98 (0.77–1.25)	-	-
25 ≤ BMI < 30 kg/m^2^	1.02 (0.80–1.32)	-	-
BMI ≥ 30 kg/m^2^	0.45 (0.27–0.75)	0.44 (0.27–0.73)	0.46 (0.28–0.77)
Hypertension	1.25 (0.93–1.67)	-	-
**RA-disease dependent risk factors**			
Symptom duration at diagnosis	0.96 (0.93 − 0.1)	0.99 (0.95–1.02)	-
RF-positivity	1.18 (0.95–1.47)	1.50 (1.16–1.81)	1.39 (1.07–1.81)
ACPA-positivity	1.05 (0.81–1.34)	1.43 (1.11–1.85)	1.30 (0.98–1.74)
DAS28			
at diagnosis	1.20 (1.10–1.31)	1.13 (1.03–1.24)	1.13 (1.03–1.24)
at 6-month	1.12 (1.04–1.21)	1.10 (1.01–1.19)	1.14 (1.04–1.25)
at 1-year	1.13 (1.04–1.22)	1.10 (1.02–1.20)	1.13 (1.04–1.24)
at 2-year	1.07 (0.99–1.16)	1.05 (0.96–1.14)	-
DAS-AUC over the 2 years, per 10	1.05 (1.00-1.10)	1.03 (0.98–1.08)	1.06 (1.00-1.11)
DAS28 ≤ 2.6 at 6-month	0.73 (0.56–0.95)	0.81 (0.62–1.06)	-
DAS28 ≤ 2.6 at 1-year	0.82 (0.65–1.05)	-	-
DAS28 ≤ 2.6 at 2-year	0.77 (0.60–0.97)	0.81 (0.64–1.04)	0.78 (0.59–1.03)
DAS28 ≤ 2.6 sustained	0.60 (0.42–0.84)	0.64 (0.46–0.91)	0.60 (0.40–0.92)
DAS28 ≤ 3.2 at 6-month	0.77 (0.71–0.96)	0.79 (0.63-1.00)	0.78 (0.60–1.02)
DAS28 ≤ 3.2 at 1-year	0.79 (0.63–0.99)	0.81 (0.65–1.02)	0.79 (0.62–1.03)
DAS28 ≤ 3.2 at 2-year	0.97 (0.77–1.21)	-	-
DAS28 ≤ 3.2 sustained	0.71 (0.55–0.92)	0.74 (0.57–0.96)	0.71 (0.51–0.98)
HAQ			
at baseline	1.01 (0.98–1.04)	-	-
at 6-month	1.46 (1.22–1.74)	1.18 (0.99–1.40)	1.23 (0.99–1.53)
at 1-year	1.42 (1.19–1.69)	1.17 (0.98–1.39)	1.35 (1.09–1.68)
at 2-year	1.45 (1.22–1.72)	1.17 (0.98–1.39)	1.17 (0.95–1.44)
HAQ ≤ 0.5 at 6-month	0.71 (0.57–0.89)	0.79 (0.63-1.00)	0.84 (0.64–1.10)
HAQ ≤ 0.5 at 1-year	0.74 (0.60–0.93)	0.81 (0.65–1.02)	0.78 (0.60–1.02)
HAQ ≤ 0.5 at 2-year	0.60 (0.48–0.76)	0.72 (0.57–0.90)	0.76 (0.58–0.99)
HAQ ≤ 0.5 sustained	0.76 (0.58–0.99)	0.74 (0.59–0.94)	0.76 (0.58-1.00)
HAQ ≥ 1 at 6-month	1.65 (1.31–2.08)	1.38 (1.09–1.75)	1.35 (1.02–1.79)
HAQ ≥ 1 at 1-year	1.60 (1.27–2.02)	1.33 (1.06–1.69)	1.44 (1.09–1.91)
HAQ ≥ 1 at 2-year	1.42 (1.12–1.82)	1.15 (0.90–1.48)	-
HAQ ≥ 1 sustained	1.54 (1.14–2.08)	1.16 (0.85–1.58)	-
**Radiographic RA-disease characteristics**			
SHS score at baseline	1.01 (1.00-1.02)	0.99 (0.98–1.01)	-
Erosion score at baseline	0.99 (0.96–1.03)	-	-
JSN score at baseline	1.02 (1.01–1.03)	1.00 (0.99–1.01)	-
SHS score at 2-year	1.01 (1.00-1.01)	1.00 (1.00-1.01)	-
Erosion score at 2-year	1.00 (0.99–1.02)	-	-
JSN score at 2-year	1.02 (1.01–1.03)	1.01 (1.00-1.01)	-
SHS progression at 2-year	1.24 (0.97–1.59)	1.04 (0.81–1.33)	-
Erosion progression at 2-year	1.26 (0.99–1.61)	1.24 (0.97–1.58)	1.37 (1.03–1.82)
JSN progression at 2-year	1.29 (1.02–1.63)	1.04 (0.83–1.32)	-
**RA-treatments**			
Daily prednisolone usage			
at baseline	1.12 (0.90–1.39)	1.23 (0.99–1.53)	1.16 (0.90–1.50)
6-month	1.03 (0.83–1.29)	1.12 (0.89–1.40)	1.15 (0.88–1.49)
1-year	1.19 (0.96–1.49)	1.27 (1.02–1.58)	1.30 (1.00-1.70)
2-year	1.20 (0.95–1.51)	1.24 (0.98–1.55)	1.20 (0.91–1.57)
Daily prednisolone dosage			
at baseline	1.01 (0.99–1.03)	1.01 (0.99–1.03)	1.01 (0.99–1.04)
6-month	1.01 (0.98–1.04)	1.02 (0.99–1.05)	1.04 (1.00-1.07)
1-year	1.02 (0.99–1.06)	1.04 (1.01–1.07)	1.05 (1.01–1.09)
2-year	1.02 (0.98–1.05)	1.043 (1.01–1.08)	1.05 (1.01–1.09)
AUC of prednisolone dosage over 1 year, per 10 mg	1.02 (0.99–1.05)	1.04 (1.01–1.06)	1.04 (1.00-1.07)*
AUC of prednisolone dosage over 2 years, per 10 mg	1.01 (1.01–1.03)	1.02 (1.01–1.04)	1.02 (1.00-1.04)*
Methotrexate over 2 years	1.22 (0.98–1.52)	1.20 (0.96–1.49)	1.21 (0.87–1.77)*
Combined cDMARDs over 2 years	1.09 (0.83–1.44)	1.49 (1.12–1.97)	1.31 (0.89–1.93)*
Biological agent over 2 years	0.87 (0.54–1.41)	-	1.05 (0.58–1.90)*

^a^Adjusted for age and sex (if appropriate), period of inclusion, BMI, smoking, prior fracture, osteoporosis, hypertension, DAS28 at baseline or *a cumulative DAS over the indicated period of observation

BMI, body mass index; RF, rheumatoid factor; ACPA, anti-citrullinated protein antibodies; DAS28, 28-joint disease activity score; HAQ, Health Assessment Questionnaire; SHS, Sharp-van der Heijde score; JSN, joint space narrowing; AUC, area under the curve; cDMARDs, conventional disease-modifying anti-rheumatic drugs

Sustained: indicated values maintained at the assessments of 6-month, 1-year and 2-year

Progression: progression of a score from baseline ≥ 1 unit

The presence of RF and erosion score progression at 2-year on fracture outcome were significantly associated with the fracture risk, and there was an indication towards an increased risk of fracture in the presence of ACPA.

The increased proportional risks of fractures with GC therapy, versus no GC, were observed at 1-year, HR 1.30 (95% 1.00 to 1.69), and with a higher GC dosage at follow-ups, 1.04 (1.00 to 1.07) at 6-month, 1.05 (1.01 to 1.09) at 1-year, and 1.05 (1.01 to 1.09) at 2-year. The risk of fracture outcome was not different by duration of daily prednisolone usage, defined as up to 6-month, 1-year or prolonged up to 2-year observation, but the estimates of fracture risk were increased with a higher cumulative exposure to GC over the first year, HR 1.04 (95% 1.01 to 1.07), as well as over the first two years, 1.03 (1.01 to 1.04). Prescription of GC and combined DMARDs is channeled towards a severely active disease. However, when cumulative disease activity was considered (Table 2), the observed risk estimates associated with cumulative exposure to GC were similar to the findings in analyses adjusted to baseline DAS28. In contrast, effects of treatments with MTX, combined DMARDs and biologics on the fracture outcome were neutral in this cohort, so the adjusted risk of fracture outcome was not statistically different by exposure to these therapies with adjustment for disease activity.

In the sensitivity analyses on outcome of a hip fracture, the observed proportional risks were consistent with the main analyses (on outcome of four major fractures) for effects of BMI at inclusion (HR 0.89, 95% 0.84 to 0.93), BMI ≤ 20 kg/m^2^ (2.79, 1.64 to 4.75), sustained DAS28-remission (0.51, 0.27 to 0.97), sustained DAS28-LDA (0.61, 0.38 to 0.99), sustained HAQ ≤ 0.5 (0.76, 0.58 to 0.99), presence of RF autoantibodies (1.39, 1.03 to 1.88), and erosion score progression at 2-year (1.72, 1.13 to 2.59).

### Time trends in occurrence of fracture

The fracture outcome occurred in 64 patients included in 1992–1995 (14.5%), in 123 patients included in 1996–1999 (15.3%), and in 165 patients included in 2000–2006 (12.6%), p = 0.176. The effect of the inclusion period on outcome was significant, log-rank Mantel-Cox p < 0.001, long-rank test for trend p < 0.001. However, in sensitivity analyses on the hip fracture outcome based on the inpatient registration and limiting the follow-up to 6 years for all inclusion periods (to account for the different follow-up times), there was no difference in outcome between patients included in 1992–1995, 1996–1999 and 2000–2006.

### BMD and occurrence of fracture

We performed a subgroup analysis in 602 patients with BMD measures at baseline, of whom, 501 patients were included in 1996–1999. The main baseline characteristics such as age, sex, smoking, BMI, prior fractures, presence of RF and ACPA, and HAQ in patients with and without BMD measures were similar, but the patients with BMD measures had a higher baseline DAS28, were treated more often with daily prednisolone and used it at a higher dosage, as well as they were treated less often with MTX and biological agents. During follow-up, major index fractures occurred in 97 of 602 patients (16.1%).

**Fig. 3:**
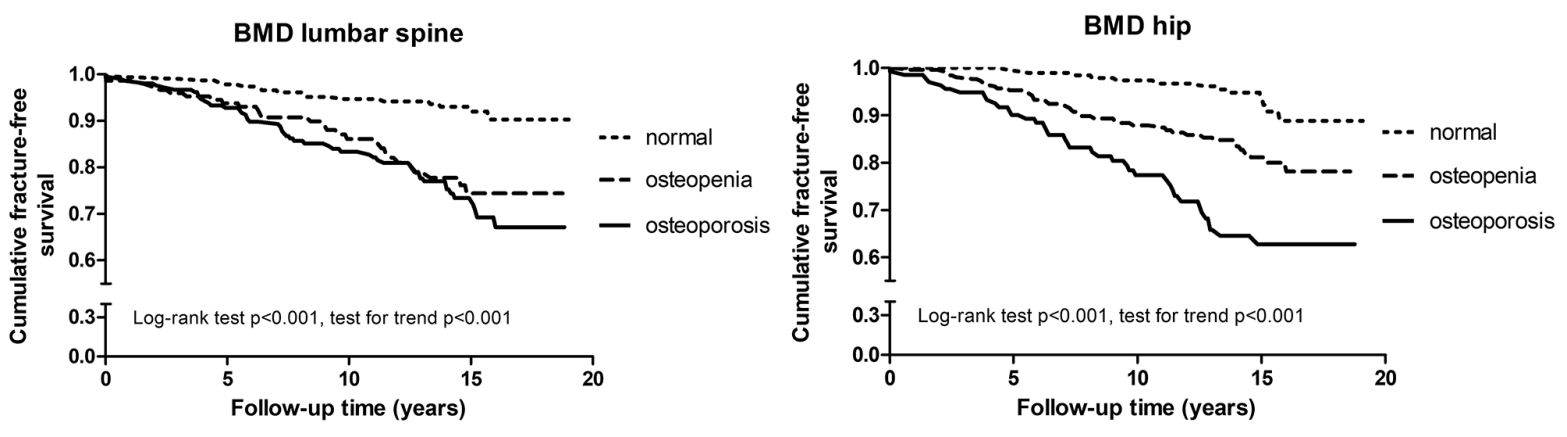
Kaplan-Meier curves of fracture-free survival in early RA, according to BMD at inclusion. Illustrates Kaplan-Meier curves of cumulative fracture-free survival in 604 patients with early RA, according to BMD at inclusion. Normal BMD was defined as the lowest T-score more than − 1.0 SD, osteopenia as a T-score − 1.0 to -2.5 SD, and osteoporosis if a T-score of -2.5 SD or less. Fracture occurrence by BMD of lumbar spine: fractures occurred in 17 out of 239 patients with BMD within the normal range, in 28 out of 212 patients with osteopenia, and in 52 out of 151 patients with osteoporosis. Fracture occurrence by BMD of hip: fractures occurred in 13 out of 202 patients with BMD within the normal range, in 42 out of 260 patients with osteopenia, and in 22 out of 140 patients with osteoporosis

Patients with a lower BMD were significantly more likely to experience a fracture than those with a normal BMD, as illustrated by Kaplan–Meier curves in Fig. 3. In Cox regression analysis adjusted for age, sex, BMI, smoking, prior fracture and DAS28 at baseline, there was an indication towards a decreased proportional risk for fracture in association with higher T-scores of lumbar spine, HR 0.87 (95% 0.75 to 1.02), and a significant risk reduction at higher T-scores of hip, adjusted HR 0.70 (0.57 to 0.86).

We questioned whether the effects of disease factors and treatments observed in the main analyses were independent of low BMD. With additional adjustment for a baseline T-score, consistently, we observed a risk reduction of fracture outcome for BMI [HR 0.93 (95% 0.88 to 0.99); 0.943 (0.89 to 0.99), respectively by adjustment for T-score of lumbar spine and hip], and DAS28-remission attained at 6-month of treatment initiation and sustained up to 2 years [0.51 (0.26 to 0.99); 0.52 (0.28–0.99)], whereas risk for fractures was increased in presence of RF autoantibodies [1.75 (1.10 to 2.80); 1.65 (1.04 to 2.62)], ACPA [1.81 (1.13 to 2.29); 1.73 (1.09 to 2.75)] and erosion score progression at 2-year [1.75 (1.07 to 2.86); 1.78 (1.10 to 2.90)]. There was, however, no difference in outcome by use of GCs, so the effects of daily GC use, daily GC dosage and cumulative exposure to GCs over 2 years on fracture outcome were not statistically significant with adjustment for BMD measures (not shown).

Because BMI could be an indicator of BMD, we further examined a possible interaction between BMI and T-scores. Indeed, there was a significant correlation between BMI and T-scores of lumbar spine and hip, respective Spearman rho coefficients of 0.25 (95% 0.17–0.33), and 0.21 (0.13–0.29), both p < 0.001. Therefore, we repeated analysis within a low range of BMD with a T-score of -1 SD or less. In this sub-analysis, the benefit of a higher BMI on the reduction in fracture was confirmed, HR 0.93 (0.87 to 0.98), and 0.93 (0.88 to 0.99), respectively for BMD of lumbar spine and hip.

## Discussion

This study in the large inception RA cohort of 2557 patients explored whether patient´s and disease characteristics at diagnosis and during the first two years after treatment initiation influenced the occurrence of future major fragility fractures of proximal femur, vertebral, proximal humeral, and distal forearm during a follow-up period of about 10 years. We show that, overall, BMI and classical disease-related risk factors, including disease activity, disability, GC therapy, antibody status and progressive erosive disease have a substantial impact on fracture outcome, independent of traditional osteoporotic risk factors. The observed effects of BMI, DAS28-remission and restored functional capacity attained at 6-month after treatment initiation and sustained up to 2-year, presence of RF, ACPA and erosive disease were independent of low BMD. The findings emphasize the importance of treat-to-target (T2T) strategy and restored function in early arthritis to reduce fracture risk.

We believe that the present data are the first to provide evidence for a reduction of fracture risk in patients who achieved sustained DAS28-remission or DAS28-LDA over the first two years of RA-disease, and in patients with a functional capacity of HAQ ≤ 0.5. A substantial risk reduction of fracture occurrence for sustained DAS28-remission and low HAQ ≤ 0.5 was consistently observed in a low BMD range. These results are important as they imply that adequate control of inflammation and normalization of functional level in the early phases of the disease may mitigate RA as a risk factor for fracture across a broad RA population, including the at-risk group of patients with bone mass deficits. These findings are in line with the reports on reciprocal regulation of bone and immune inflammatory cells, and the interference of systemic inflammation with bone remodeling, uncoupling bone resorption and bone formation [27–29]. Treatment of RA with medications that inhibit specific inflammatory cytokines, such as interleukin (IL-6) and tumor necrosis factor alpha (TNFa), has been shown to prevent bone loss, remarkably both in responders and non-responders, and to improve BMD in some studies of experimental design [30–32]. In the studies based on large administrative databases, risk of fragility fractures has been comparable in patients treated with biological DMARDs and non-biological DMARDs in prevalent cases of RA and arthritic diseases [33, 34]. Also, in our observational cohort of early RA, treatments with MTX, combined DMARDs and biological DMARDs during the first two years after diagnosis resulted in similar effects on the fracture outcome, when adjusted for disease activity. To date, the only known mechanism for an effect of anti-rheumatic treatment on bone health is through the inflammatory pathway, which is likely to be more pronounced using an early T2T approach during the presumed therapeutic “window of opportunity”. Of note, during the first two-year period of RA-disease the annual rate of bone loss was higher than that in the following 2–10 year period of follow-up in early RA patients, and treatment variables have been more frequently found to be associated with bone loss than well-known risk factors for osteoporosis, which supports the importance of early aggressive anti-inflammatory treatment [35]. BMD in RA patients in remission and controls without RA has not been different [36], supporting a dissociation between RA and reduction of BMD if disease activity is controlled. The findings of our analysis and other studies suggest that control of disease activity in early RA with any DMARDs, non-biological or biological, can lead to important benefits on bone health and can lower the fracture risk.

A low BMD and more fractures have been shown in RA patients with poor functional status [7, 37], which is likely to be multifactorial and could be regarded as a summary measure of the overall impact of the disease. The relation between functional disability and increased risk of fractures was confirmed in our study. Of note, although the HAQ score was substantially elevated at diagnosis in our patients, a significant fracture risk reduction was observed here in those with restored functional status at 6-month after treatment initiation and sustained up to two years. Thus, assessment of physical function and interventions aiming to reverse disability in early RA could be important for fracture prevention in RA.

Although RA treatment strategies in the last years have advanced towards early treatment and tight control targeted at sustained remission, fragility fractures are still more frequent in RA than in non-RA populations [8, 38]. An increasing rate of hip fractures at an earlier average age than in the general population has even been reported in the aging RA population [39]. However, uncertainty regarding diagnostic accuracy is a concern in studies that identify cases from administrative databases through an extended period. In this regard, the finding of a significant effect of the inclusion period on the fracture outcome in our analysis should be treated with caution, because the possibility of confounding due to underestimation of fractures at some sites in the more past years could not be excluded. Our sensitivity analysis restricted to hip fractures and with consideration to time exposure for each inclusion period did not confirm a modification of outcome by inclusion period, but low numbers could have led to false-negative results. To arrest generalized bone loss in early RA and to reduce a future fracture risk, a comprehensive treatment integrating osteoporosis specific strategies could be needed and warrants further study.

GC therapy is associated with well-known toxicities, particularly at high doses. The delayed toxicity of low-dose GC therapy is likely to reduce the potential benefits of symptoms relief, but the benefit/risk ratio is still controversial [40]. A recent study has shown that GC use in RA, even at low doses, was associated with an elevated risk of osteoporosis and other important comorbidities such as diabetes mellitus, thrombotic stroke, myocardial infarction, serious infection and death [41].

Our study provides additional evidence for bone toxicity with GC therapy with a significant fracture risk elevation in patients on daily GC at follow-ups, exposed to higher cumulative dose of GC over two years after RA treatment initiation. The observed excess risk was independent of disease activity and traditional risk factors of osteoporosis, which argues against the perceived positive benefit/risk ratio of GC and inclusion of GC in therapeutic strategy in early RA. A study in new-onset RA has reported a significantly elevated fracture risk at high levels of daily and cumulative GC dose even in relatively young RA population of 18–64 years aged [42]. In our study, the exposure to GC does not appear to confer an excess in fractures when bone mass is considered, suggesting that this risk excess in relation to GC therapy would be most applicable to RA patients with bone mass deficits. It is however important to emphasize that bone density data cannot directly be translated to fracture risk in patients treated with GC because patients using GC may have a higher risk of fracture independent of the BMD level.

As to RA-specific markers, the presence of RF, ACPA and progression of erosion score indicated an increased risk of fractures in our analysis, also when bone mass was considered. Therefore, these factors could be applicable for risk stratification in RA patients across all BMD measures. The effect of RF and erosion progression on risk of fracture was consistently significant when disease activity was considered. Therefore, fracture prevention strategy could be of particular benefit in RF-positive patients and in progressively erosive disease, also when disease activity is controlled. Given a trend significant finding for the ACPA effect, additional confirmation is needed to resolve whether ACPA contributes to fracture risk in well-treated disease. Although pre-clinical studies provide some insight into both inflammation-dependent and inflammation-independent interaction of autoantibodies related to RA with bone cells [43], and that early induction of osteoclast differentiation and activation could be autoantibody mediated and starts even before RA onset [44], few clinical studies examined the role of autoantibodies for bone health. Our study reports an excess risk of fractures in the presence of RF and early progressively erosive RA, a risk which might be uncoupled from inflammation. Relevant to this question, previous studies have reported significant associations between ACPA positivity and high RF antibody levels with lower systemic BMD in patients with early RA, as well as a higher FRAX score in ACPA positive RA patients [4, 15, 44, 45]. Autoantibody positivity is considered as a specific risk factor also for a periarticular bone loss and erosive RA. Progressively erosive disease can reflect persistent inflammation and/or additional immune pathways triggering osteoclasts activity and inhibiting osteoblast function. Therefore, it could be hypothesized that erosive disease (local bone loss) is associated with systemic bone loss, which, in turn, could be associated with risk of fractures. Indeed, presence of vertebral fractures has been associated with long disease duration, CRP and Sharp erosion score in patients with established RA [46], while correlation between Sharp erosion score and BMD was not significant on multiple adjustments in other study [47]. Interestingly, bisphosphonates and anti-RANKL therapy combined with DMARDs could slow down erosion progression, but not cartilage loss, in early RA [48, 49], which support the hypothesis of interplay between local and generalized bone loss in RA.

Besides systemic inflammation, general risk factors for osteoporosis are of importance in defining fracture risk in RA. Our data confirm the benefit of a higher BMI and obesity on the reduction in fracture risk. The phenomenon of “the obesity paradox” is thought to be determined by the cross-talk between the bone tissue and the adipose tissue, and the association between higher BMD and higher BMI is well known. We found however reductions in fracture risk with a higher BMI also in a low BMD range, suggesting that the magnitude of the excess of fracture risk in low BMD would likely be smaller in overweight in comparison with the generally reported increased fracture risk in patients with bone mass deficits. Concerning the global risk of fracture, both protective and harmful effects of BMI have to be considered, including a substantially increased fracture risk in low weight patients, also confirmed in our cohort.

Some limitations of our analysis warrant consideration. The limitations of the present study include those that cannot be ignored in observational cohort studies, with potential confounding factors which could not be taken into account. We used a composite endpoint, thence, additional confirmation is needed for a particular fracture site. Vertebral fractures might be asymptomatic with potential risk of underestimation without additional diagnostics. Baseline BMD data were available only from a proportion of included RA patients, however, we found the same pattern and range of the estimated risk of the fracture outcomes in the subgroup analysis as in the main analysis. The effect of GC therapy over the entire time period until a fracture event deserves further research.

Our study has a number of strengths. The BARFOT cohort allows exploration of the outcome in a large non-selected RA patient population, including both women and men, followed prospectively after the diagnosis and treated in ordinary daily clinical practice in the end of the old and in the new millennium. Follow-up time was sufficiently long and consistent with the time-horizon of prediction validated for the FRAX tool. Diagnosis of fractures was based both on medical records and hospital discharge registers.

## Conclusions

This large cohort study in early RA provides evidence for the effects of several RA specific characteristics over the first two years after treatment initiation on fracture risk over about 10 years. Our findings suggest that for fracture prevention in patients with RA implementation of comprehensive strategy is needed based on (i) T2T strategy and normalization of function in early phases of RA-disease, (ii) additional personalized strategies in patients at-risk of fractures, such as autoantibody positivity, progressively erosive disease, and low body weight, (iii) and appropriate assessment of BMD.

## Data Availability

The datasets analyzed in the current study are available from the corresponding author on reasonable request. Individual participant data will not be made available.

## List of abbreviations

ACPA: Anti-citrullinated protein antibodies
ACR: American College of Rheumatology
ANOVA: Analysis of variance
AUC: Area under the curve
BARFOT: Better Anti-Rheumatic PharmacO Therapy
BMD: Bone mineral density
BMI: Body mass index
CI: Confidence interval
CVD: Cardiovascular disease
DAS28: 28-joint disease activity score
DMARDs: Disease-modifying anti-rheumatic drugs
DXA: Dual-energy x-ray absorptiometry
FRAX: Fracture Risk Assessment tool
GC: Glucocorticoids
HAQ: Health Assessment Questionnaire
HR: Hazard ratio
ICC: Intraclass correlation coefficient
ICD: International Classification of Diseases
IL: Interleukin
IQR: Interquartile range
JSN: Joint space narrowing
LDA: Low disease activity
MTX: Methotrexate
NSAIDs: Non-steroidal anti-inflammatory drugs
RA: Rheumatoid arthritis
RF: Rheumatoid factor
SD: Standard deviation
SHS: Sharp–van der Heijde score
T2T: Treat-to-target
TNFa: Tumor necrosis factor alpha
